# Pre-treatment vitamin B12, folate, ferritin, and vitamin D serum levels in patients with warts: a retrospective study

**DOI:** 10.3325/cmj.2020.61.28

**Published:** 2020-02

**Authors:** Funda Tamer, Mehmet Eren Yuksel, Yavuz Karabag

**Affiliations:** 1 Department of Dermatology, Gazi University School of Medicine, Ankara, Turkey; 2 Department of General Surgery, Aksaray University School of Medicine, Aksaray, Turkey; 3 Department of Cardiology, Kafkas University School of Medicine, Kars, Turkey

## Abstract

**Aim:**

To compare the serum levels of 25-hydroxyvitamin D, ferritin, folate, vitamin B12, zinc, and thyroid stimulating hormone between patients with warts and healthy individuals.

**Methods:**

This retrospective study enrolled 40 patients with warts and 40 healthy individuals treated at the Ufuk University Hospital, Ankara, between July and December 2017. Serum levels of 25-hydroxyvitamin D, ferritin, folate, vitamin B12, zinc, and thyroid stimulating hormone status were evaluated retrospectively.

**Results:**

Participants with and without warts had similar mean serum 25-hydroxyvitamin D, ferritin, folate, zinc, and thyroid stimulating hormone levels. However, patients with warts had significantly lower mean serum vitamin B12 level (*P* = 0.010). Patients with warts non-significantly more frequently had decreased serum levels of 25-hydroxyvitamin D, ferritin, and folate (*P* = 0.330, *P* = 0.200, *P* = 0.070, respectively).

**Conclusion:**

Patients with warts may require evaluation of serum levels of vitamin B12, folate, ferritin, and vitamin D.

Warts are benign epithelial proliferations caused by human papillomavirus infections (1-3). They are classified as common, plantar, flat, filiform, and genital (4). Common warts can occur at any part of the skin depending on host immunity (2), but mostly on hands and knees. Warts are usually treated with topical and systemic immunotherapy. Most frequently used immunotherapy agents are topical imiquimod, bacillus Calmette-Guérin vaccine, human papillomavirus vaccine (5,6), intralesional interferon-α2b and interferon-β, systemic zinc, cimetidine, and levamisole (7). Other treatment options include topical salicylic acid, 5-fluorouracil and glutaraldehyde therapy, cryotherapy, excision, electro**-**cauterization, and laser ablation (8,9). The treatment success depends on the virus type, duration and number of warts, and host’s immune status (10). However, warts are usually resistant to treatment, especially in adults and immunosuppressed patients (8-10) and no single therapy has been considered as a gold standard (8,9).

Cutaneous and genital warts have been successfully treated by the topical application of vitamin D3 derivatives and intralesional vitamin D3 injections (7,11-13). Vitamin D3 derivatives play a role in the regulation of epidermal cell proliferation, differentiation, and cytokine production (11). Other micronutrients, such as zinc, iron, folate, vitamins A, C, E, B6, B12, and thyroid hormones, have also been reported to regulate immune response (14-16). Al-Gurairi et al (17) suggested that oral zinc sulfate might be an effective therapeutic option in the treatment of recalcitrant warts. We, therefore, hypothesized that patients with warts had decreased levels of these micronutrients and hormones. The aim of this study was to compare the serum levels of vitamin D, ferritin, folate, vitamin B12, zinc, and thyroid stimulating hormone (TSH) in patients with cutaneous and genital warts and healthy individuals.

## PATIENTS AND METHODS

This study enrolled 40 patients with warts and 40 healthy individuals who were admitted to Ufuk University Hospital dermatology outpatient clinic between July 2017 and December 2017. The study was approved by the Ufuk University Ethics Committee (20171101-4), and all participants gave informed consent. Medical records were reviewed retrospectively.

The inclusion criterion for the patient group was having any type of viral warts. All patients with warts regardless of age, sex, and race were included to avoid selection bias. Warts were diagnosed based on dermatological examination. The exclusion criteria were pregnancy, lactation, metabolic and endocrine disorders, dermatological diseases other than warts, malignancy, hematologic disorders, inflammatory bowel diseases such as ulcerative colitis and Crohn’s disease, gastrointestinal surgery, atrophic gastritis, chronic liver diseases, chronic kidney diseases, hormone-replacement therapy, chemotherapy, immunosuppressive therapy, vitamin and mineral supplements, eating disorders, and diet restrictions. Patients who had lesions with atypical clinical appearance were also excluded. The control group consisted of age and sex-matched healthy individuals who did not have warts or any disorders stated as exclusion criteria. They were admitted to our department for routine dermatological examination and wanted to check their vitamin status. The localization of the warts, clinical type of the warts, lesion number, disease duration, symptoms like pain or itching, skin phototype, previous treatments, and medical history and family history were recorded. Serum levels of ferritin, folate, vitamin B12, zinc, TSH, and 25-hydroxyvitamin D (25(OH)D) were evaluated.

### Statistical analysis

Normality testing was conducted with the Kolmogorov-Smirnov test. Continuous data are expressed as mean and standard deviation or median and interquartile range. The significance of difference between the groups was assessed with the *t* test or Mann-Whitney U-test and analysis of variance or Kruskal-Wallis, where applicable. The categorical variables are expressed as counts and percentages, and significance of difference between the groups was assessed with the Fisher exact test or χ^2^-test. The level of statistical significance was set at *P* < 0.05. Statistical analysis was performed with SPSS version 22.0 (IBM Corp., Armonk, NY, USA).

## RESULTS

Participants with and without warts did not significantly differ in sex (26 women and 14 men in each group), age (31.6 ± 14.4 years and 29.2 ± 11.4 years, respectively), and Fitzpatrick score. Thirty-four patients (85%) had Fitzpatrick skin type III and 6 (15%) patients had Fitzpatrick skin type IV. Thirty-five (87.5%) controls had Fitzpatrick skin type III and 5 (12.5%) had Fitzpatrick skin type IV. Patients’ characteristics are shown in Table 1. The medical history of 37 (92.5%) patients was unremarkable, whereas 3 (7.5%) patients had hypertension. Only 1 (2.5%) patient reported that her brother also had cutaneous warts. The controls' medical history was unremarkable.

**Table 1:** Characteristics of patients with warts

Characteristic	No. (%) of patients
Wart type	
warts on hands	11 (27.5)
plantar warts	21 (52.5)
anogenital warts	8 (20)
Number of lesions	1 to 16
Mean disease duration (±standard deviation), months	7.7 ± 8.6
Symptoms	
no symptoms	30 (75)
pain	10 (25)
Previous treatment	
none	23 (57.5)
cryotherapy	9 (22.5)
topical	7 (17.5)
electro**-**cauterization	1 (2.5)
Treatment at our institution	
cryotherapy	35 (87.5)
electro**-**cauterization	2 (5)
topical	3 (7.5)

Patients and controls had similar mean serum 25(OH)D, ferritin, folate, zinc and TSH levels. However, patients had significantly lower mean serum vitamin B12 level compared with participants without warts (*P* = 0.010). Patients non-significantly more frequently had decreased levels of serum vitamin B12, 25(OH)D, ferritin, and folate (*P* = 0.130, *P* = 0.330, *P* = 0.200, *P* = 0.070, respectively) (Table 2 and Table 3).

**Table 2:** Micronutrients and thyroid stimulating hormone in patients with and without warts*

	Patients with warts	Patients without warts	P
Vitamin B12 (pg/mL), mean ± standard deviation	271.4 (94.3)	341.4 (127.8)	0.010
Folate (ng/mL), mean ± standard deviation	6.6 (3.2)	6.2 (2.2)	0.640
25-hydroxyvitamin D (ng/mL), mean ± standard deviation	16.69 (6.49)	16.35 (9.83)	0.390
Zinc (μg/dL), mean ± standard deviation	92.2 (25.1)	82.7 (16.6)	0.110
Ferritin (ng/mL), median and range	37.27 (1.71-301.17)	27.89 (2.51-156.94)	0.650
Thyroid stimulating hormone (μIU/mL), median and range	1.75 (0.31-6.75)	1.93 (0.1-3.41)	0.800

**Table 3:** Micronutrients and thyroid stimulating hormone in patients with and without warts

	Patients with warts			Patients without warts		P
	low	normal	high	low	normal	
Vitamin B12	9 (22.5)	31 (77.5)	0 (0)	4 (10)	36 (90)	0.130
Folate	3 (7.5)	37 (92.5)	0 (0)	0 (0)	40 (100)	0.070
25-hydroxyvitamin D	30 (75)	10 (25)	0 (0)	26 (65)	14 (35)	0.330
Zinc	1 (2.5)	39 (97.5)	0 (0)	1 (2.5)	39 (97.5)	1.000
Ferritin	8 (20)	32 (80)	0 (0)	3 (7.5)	37 (92.5)	0.200
Thyroid stimulating hormone	1 (2.5)	38 (95)	1 (2.5)	1 (2.5)	39 (97.5)	0.560

Patients with warts on hands, patients with plantar warts, and patients with genital warts were separately compared with healthy individuals in terms of serum levels of 25(OH)D, ferritin, folate, TSH, vitamin B12_,_ and zinc. Patients with warts on hands significantly more frequently had decreased serum folate (2/11 or 18.2% vs 0%, *P* = 0.006), patients with plantar warts had significantly lower serum vitamin B12 (254.2 ± 104.6 pg/mL vs 341.4 ± 127.8 pg/mL, *P* = 0.005), and patients with genital warts significantly more frequently had decreased serum 25(OH)D (8/8 or 100% vs 26/40 or 65%, *P* = 0.049).

## DISCUSSION

In our study, patients with warts had significantly lower mean serum vitamin B12 level than patients without warts. Furthermore, they more frequently had decreased serum vitamin B12 levels. Patients with plantar warts had significantly lower mean serum vitamin B12 level than patients without warts. Therefore, we suggest that patients with warts should be assessed for serum vitamin B12 levels. Vitamin B12 enhances T cell proliferation and immunoglobulin synthesis, and its lack may decrease the protective immune responses to viruses and bacteria (18). Hu et al (19) successfully treated flat warts by acupuncture point injection of vitamin B12.

Patients with warts and healthy individuals did not significantly differ in serum 25(OH)D levels. However, the number of participants with low 25(OH)D levels was greater in the group with warts (75%) than in the group without warts (65%). Furthermore, low serum 25(OH)D level was significantly more prevalent in patients with genital warts (100%) compared with healthy individuals (65%). Therefore, we suggest that serum levels of vitamin D should be checked in patients with warts, and vitamin D supplementation should be advised when needed. Topical vitamin D derivatives have been successfully used in warts treatment (11-13,20,21) but also in the treatment of seborrheic keratosis, psoriasis, transient acantholytic dermatosis, actinic porokeratosis, and keratosis palmaris et plantaris (12,22). They play a role in the regulation of epidermal cell proliferation, differentiation, and cytokine production (11), inhibit hyperkeratosis and inflammation, and induce apoptosis (12). Moreover, oral calcitriol with a daily dose of 0.5 μg reduced lesions in patients with widespread seborrheic keratosis by acting antiproliferatively on keratinocytes (23,24). Most of the immune system cells express vitamin D receptor both in the resting and active phase, and vitamin D regulates immune system cell signaling and stimulates the native immune defense system. Therefore, vitamin D deficiency has been associated with an increased risk of bacterial and viral infections (25).

Patients and healthy individuals in this study had Fitzpatrick skin type III or IV. Fitzpatrick classification (type I to VI) is based on skin color and tanning and burning response to sun exposure (26). Since melanin absorbs UV radiation, individuals with Fitzpatrick skin type V have a decreased cutaneous production of vitamin D and are prone to vitamin D deficiency compared with lighter skinned population (27). This is why it is advisable to compare vitamin D status between participants with similar skin types.

The immune response is mostly influenced by the status of micronutrients, such as zinc, selenium, iron, copper, vitamins A, C, E, B6, B12, and folic acid (14,18). For instance, zinc deficiency has been associated with impaired wound healing and impaired lymphocyte and phagocyte functions (14). Low serum zinc level was more prevalent in patients with resistant warts lasting more than six months than in controls, suggesting a possible association of zinc deficiency with persistent, progressive, or recurrent viral warts (28).

Folate deficiency may lower the resistance to infections by decreasing T lymphocyte proliferation, while folate supplementation could improve the immune response (15). A previous study showed that decreased serum folate levels were implicated in cervical infections with high-risk HPV types (29). However, this is the first study to our knowledge that assessed the association between serum folate levels and common warts.

Innate and adaptive immunity are also affected by thyroid hormones. The relationship between thyroid hormones and immune cells is complex, but hypothyroidism was associated with decreased lymphocyte function (16). Iron is an essential nutrient with functions in various cell processes (30). Both iron deficiency and iron excess can influence the innate and adaptive immune system functions. Iron deficiency has been reported to lead to increased susceptibility to infections (31).

The limitations of this study were small sample size, low statistical power of the non-significant results, and retrospective study design. Despite these limitations, we believe that this study is a useful contribution to the body of knowledge on the role of micronutrients and hormones in patients with warts.

In conclusion, we suggest that in addition to vitamin B12 assessment, patients with warts should also be assessed for serum levels of 25(OH)D, folate, and ferritin. As these micronutrients and vitamins play an important role in immune response, supplements may help in warts treatment. However, further studies with larger sample size are needed both to confirm these results and monitor the outcomes in a longer follow-up period.
